# Effect of Grafted Insect Protein with Palatinose on Quality Properties of Phosphate-Free Meat Emulsion

**DOI:** 10.3390/foods11213354

**Published:** 2022-10-25

**Authors:** Tae-Kyung Kim, Yea-Ji Kim, Jake Kim, Hyun-Jung Yun, Min-Cheol Kang, Yun-Sang Choi

**Affiliations:** Research Group of Food Processing, Korea Food Research Institute, Wanju 55365, Korea

**Keywords:** phosphate substitutes, grafting technology, insect protein, meat emulsion

## Abstract

Due to concerns about the negative effects of phosphate on human health, the development of phosphate substitutes is an active area of research. Among the various methods, the structural modification of proteins has previously been established. In this study, we used grafting technology. Extracted insect protein was grafted with palatinose (GI), and 0.1 and 0.15% of GI were added to a phosphate-free meat emulsion mixed with 0.1% of eggshell powder (ES). The pH, myofibrillar protein solubility, and apparent viscosity increased with the addition of GI and ES (*p* < 0.05). Color values were also affected by GI and ES addition (decreased CIE L* and CIE a* and increased CIE b*; *p* < 0.05), while cooking loss was only improved by the addition of ES and not GI. Although the total fluid separated more than negative control (*p* < 0.05), the addition of ES improved emulsion stability and total expressible fluid separation and the fat separation reduced with addition of GI and ES (*p* < 0.05). Lipid oxidation was inhibited by the addition of GI and ES (*p* < 0.05). Moreover, the protein molecular weight distribution under 20 kDa was modified by the addition of GI, and the hardness and springiness of treatments decreased. In conclusion, the addition of GI and ES might be used to improve cooking loss, emulsion stability, and antioxidants, while the textural properties should be further researched.

## 1. Introduction

Phosphate, along with table salt, is one of the most important additives in processed meat products [1]. Phosphate was first used in processed meat products in the 1950s, and numerous studies have been conducted on its effects [2]. The purpose of using phosphate, a synthetic additive, is to improve texture and inhibit lipid oxidation and the growth of microorganisms [3,4]. It has also been reported that adding phosphate to meat products increases pH and improves water-holding capacity, emulsion stability, and processing yield [1,4]. The mechanism of action of phosphate additives in meat products includes an increase in ionic strength due to the increase in pH and water-holding capacity by dissociating actomyosin via the inactivation of calcium present in myofibrillar protein and the expansion of the water-binding space [4]. Therefore, phosphate is added to improve the quality of meat products. However, due to reports that phosphate is harmful to human health, the amount of phosphate to be used as an additive should be kept to a minimum or avoided altogether for phosphate-free products. Few technologies can replace phosphate for its ability to increase the water-holding and emulsification capacity. The trend of clean labels replacing synthetic food additives is expanding in the global market.

Edible insects have been studied as protein materials in terms of future food security [5,6]. In particular, several studies have been conducted on materials, composed of low-molecular-weight, that can replace the traditional meat proteins [1,7,8]. However, consumption of edible insects has not yet increased because of the external disgust it triggers among consumers; therefore, edible insects are powdered and used as additives. This study aimed to investigate the use of edible insect proteins as an alternative to phosphate.

As novel protein resources, the protein functionality of edible insects should be improved, and various studies have shown that the addition of edible insects in meat emulsion can decrease the quality properties of emulsified food [9,10,11]. The protein–saccharide grafting and Maillard reactions are significant chemical reactions that can enhance functional properties [12,13]. Some researchers have reported that the protein–saccharide grafting reaction is beneficial for antimicrobial, antioxidant, gelling, and emulsifying properties [14,15,16]. Kim et al. [12] reported that the myofibrillar protein isolate-saccharide graft reaction improves the quality characteristics of low-salt concentration meat products. Therefore, it can be a way to improve the functional properties of edible insect protein.

Therefore, this study aimed to evaluate the effect of a combination of grafted edible insect protein and eggshells as a phosphate replacement on the optimal quality characteristics of phosphate-free meat emulsions.

## 2. Materials and Methods

### 2.1. Materials

*Protaetia brevitarsis* larvae were obtained from a local farm (Wanju, Korea), and fresh pork ham (48 h postmortem) and back fat were purchased from a local butcher shop (Wanju, Korea). Isomaltulose, called palatinose, and sodium polyphosphate, were obtained from ES Food (Gunpo, Korea), and eggshell powder was obtained from Edentown F&B (Incheon, Korea). All chemicals were obtained from Sigma-Aldrich (St. Louis, MO, USA), except for electrophoresis (Bio-Rad Laboratories, Hercules, CA, USA).

### 2.2. Preparation of Grafted Insect Protein and Manufacturing of Meat Emulsion

A hundred grams of defatted insect powder was dissolved in 300 mL distilled water and fully hydrated for 12 h. After centrifugation at 15,000× *g* for 30 min to extract the protein, the protein concentration was regulated at 30 mg/mL [17]. Protein solution and palatinose (10 mg/mL) was homogenized well and incubated at 37 °C for 8 h to graft proteins and reduce sugars. The incubated sample was spray-dried and the powder was stored at 4 °C until it was added to the meat emulsion [15].

After removing excess connective tissue and fat, pork meat and back fat were ground through a 3 mm plate at 10 °C. Ground pork meat (500 g), salt (1.5% of total amount meat, ice, and fat), and other additives were homogenized for 1 min using a bowl cutter (C4VV, Sirman, Marsango, Italy) at the highest level of knife speed (2500 rpm). When mixed, meat, salt, phosphate, grafted insect protein, and eggshell powder were homogenized together, and treatment was determined according to the composition of these additives. The basic formulation of controls and treatments is presented in Table 1. The percentages of additives were based on the total amount of meat, ice, and fat. Flaked ice (250 g) was added to the bowl and homogenized for 1 min at the same knife speed, and then ground fat (250 g) was homogenized together with the ice for 1 min at the same knife speed [18]. The 25 g of meat emulsion was stuffed into conical tubes and heated at 75 °C for 30 min in water bath (JSSB-30T, JS Research, Gongju, Korea). Heated samples were cooled at 20 °C for 1 h to use in further experiments.

### 2.3. pH

The pH values of the homogenized raw and heated samples were estimated using a pH meter (Model 340, Mettler Toledo GmbH, Schwerzenbach, Switzerland) calibrated with a pH buffer (pH 4, 7, and 10) at 20 °C. Two grams of the sample was homogenized with 20 mL of distilled water, and the pH value of the homogenate was measured.

### 2.4. Instrumental Color

CIE L*a*b*color space was used according to the International Commission on Illumination (CIE). A CR-400 Chroma Meter (Konika Minolta, Osaka, Japan) was used, and the specific conditions were D65 illuminant, 2° observers, and Ø8 mm aperture. A white calibration plate (lightness, 97.83; redness, −0.043; yellowness, 1.98) was used for calibration. The surface of samples was measured at 20 °C.

### 2.5. Protein Solubility

Protein solubility of the raw samples was determined according to the method described by Yong et al. [19]. The raw sample was homogenized and diluted 10 times using different phosphate buffers, and the soluble protein concentration was measured using the BCA protein assay. Phosphate buffer A was used to determine total protein solubility and its composition was 1.1 M potassium iodide in 0.1 M potassium phosphate (pH 7.4). Phosphate buffer B (0.025 M potassium phosphate, pH 7.4) was used to determine sarcoplasmic protein solubility and its composition. Different protein concentrations between total and sarcoplasmic proteins were considered to be a product of myofibrillar protein solubility.

### 2.6. Sodium Dodecyl Sulfate Polyacrylamide Gel Electrophoresis (SDS-PAGE)

The protein molecular weight distribution of the raw samples was estimated using SDS-PAGE. The 1 mg/mL of protein dissolved in 1.1 M potassium iodide in 0.1 M potassium phosphate (pH 7.4) was used and 20 μg of protein was reacted with reducing sample buffer at 100 °C for 5 min. Mini-PROTEIN^®^ TGXTM 12% gel was used, and electrophoresis was performed at 100 mA. Coomassie brilliant blue R-250 staining solution was used to stain proteins, and Coomassie brilliant blue R-250 destaining solution was used to destain [20].

### 2.7. Apparent Viscosity

The apparent viscosity of the raw sample was estimated using DV3THB (Brookfield Engineering Laboratory, Middleboro, MA, USA). The samples (5 mL) were poured into a standard cylinder, and measurements were performed by continuously maintaining the same rotational speed of the spindle. The probe number was SC4-28 and temperature of sample was regulated at 15 °C. The test speed and time were 10 rpm and 35 s, respectively, and the final unit was Pa∙s.

### 2.8. Cooking Loss

The weight change after heating was calculated as cooking loss. The 25 g of raw sample was stuffed into a conical tube and heated at 75 °C for 30 min. Heated samples were taken out carefully and cooled to 20 °C for 1 h. After the released water was wiped off using a paper towel, the weight of the cooled sample was measured.

### 2.9. Water Holding Capacity (WHC)

The WHC of cooked meat emulsions was estimated using the centrifugation method [21]. The moisture content of the cooked samples was measured according to the AOAC [22]. The moisture content of the cooked samples was measured after centrifugation at 1000× *g* for 10 min. The remaining moisture content (%) was calculated as the weight difference between the moisture content of the cooked sample and that after centrifugation.

### 2.10. Emulsion Stability

The 20 g of raw meat emulsion was stuffed into ruled glass and cooked at 75 °C for 30 min. After cooling to 20 °C for 1 h, hydrophilic and hydrophobic layers were separated. The total expressible fluid (hydrophilic layer) and fat separation (hydrophobic layer) were compared with the weight of the sample and presented as a percentage [23].

### 2.11. Lipid Oxidation

Lipid oxidation of meat emulsions during heating was estimated using Thiobarbituric acid reactive substances (TBARS) methods [24]. A total of 10 g of the sample was homogenized with 100 mL of 0.1 N HCl and 5 mL of distillate was reacted with 5 mL of 0.02 M 2-thiobarbituric acid in 90% acetic acid at 100 °C for 35 min. Absorbance of the cooled sample was measured at 584 nm using a spectrophotometer (Spectra Max Plus 384, Molecular Devices Inc., San Jose, CA, USA).

### 2.12. Texture Profile Analysis (TPA)

The cooked sample was cut into sections of 10 mm in height and 25 mm in diameter before performing TPA. TA-XTplus (Stable Micro System Ltd., Surrey, England) was used, and pre-test speed, test speed, post-test speed, strain, interval time between compression cycle, data acquisition rate and trigger force were 5 mm/s, 2 mm/s, 5 mm/s, 50%, 2 s, 200 s^−1^, and 5 g, respectively [25]. The probe diameter was 35 mm, and the compression tests were performed. Eight technical replicates were conducted for each sample.

### 2.13. Statistical Analysis

The data were analyzed using three different independent batches. The tables present the mean and standard error values. A minimum of three replicates were performed for each experiment. SPSS version 20 (IBM Corp., Armonk, NY, USA) was used, and a one-way analysis of variance was performed. Significant differences in pH, color, cooking loss, WHC, emulsion stability, lipid oxidation, TPA among treatments were determined using Duncan’s multiple range test (*p* < 0.05).

## 3. Results and Discussion

### 3.1. pH and Color

The pH value of a protein affects its functional properties such as protein solubility, emulsifying and gelation properties [26]. A significant difference was observed in the pH values among the treatments (Table 2). In this study, the highest pH of the raw meat emulsion was observed at T2 (*p* < 0.05). This increased pH value may have been due to the calcium carbonate in the ES [27]. NC, which had no phosphate, had the lowest pH value, followed by T3 (*p* < 0.05). Although the effect of the amount of grafted insect protein on pH before heating was different, there was no significant difference between T1 and T3 and T2 and T4 (*p* > 0.05). In meat emulsions, increased pH due to phosphate could affect the protein net charge, and WHC and emulsifying properties could be enhanced [26]. In this study, treatments had higher pH values than NC, which might have affected the quality properties of meat emulsions such as WHC and emulsifying properties [26].

The color values of the meat emulsions containing grafted insect proteins and eggshells are presented in Table 2. The CIE L* (lightness) values can be changed by food ingredients and additives [7]. Among the raw samples, PC had the highest CIE L* value (*p* < 0.05). With the addition of GI and ES, the treatments had a lower value than PC (*p* < 0.05). These color changes were similar to those observed for CIE a* (redness) and CIE b* (yellowness). The highest value of CIE a* was observed in NC, and treatments had a lower value than NC (*p* < 0.05). When comparing CIE b* values, PC and NC had the lowest values, and T3, which had the highest GI content, had the highest value (*p* < 0.05). This result might have been due to melanin pigments in the insect extract and eggshell, which had a white color, and that could have decreased CIE b* [7,27]. Roncolini et al. [28] studied the effect of mealworm powder on the color of bread and the color also affected by typical color of mealworm. In addition, ES affected increased CIE L* and decreased CIE b* in other studies [27,29]. As described, CIE L* and CIE b* might be affected by GI and ES.

### 3.2. Protein Solubility

Protein solubility is a key characteristic of structured and stable meat emulsions [26]. Sarcoplasmic and myofibrillar proteins are typical proteins that compose porcine muscle, and these proteins can determine the textural and emulsifying properties of meat emulsions [30]. The solubility of the total, sarcoplasmic, and myofibrillar proteins is presented in Table 3. T3 and T4 had the highest total protein solubility values (*p* < 0.05), followed by T1 and T2 (*p* < 0.05). The addition of GI increased total protein solubility, and there was no significant difference among treatments, including the control groups, in sarcoplasmic protein (*p* > 0.05). Therefore, GI may affect myofibrillar proteins in meat emulsions. According to Kim et al. [7], structurally modified insect proteins can change the structural characteristics of myofibrillar proteins, and actin proteins might interact with insect proteins. In this study, myofibrillar protein solubility changed with the addition of GI. Greater addition of GI resulted in higher myofibrillar protein solubility, which might be due to increased residue length by palatinose [15]. The grafted protein has more flexible structural characteristics, and protein solubility can be enhanced owing to increased flexibility [15,16,31]. However, myosin protein plays a major role in the formation of heat-induced gels during heating [26]. Therefore, the interaction of actin with GI could inhibit myosin interaction, which can form tight and elastic gels.

### 3.3. Protein Molecular Weight Distribution

**Figure 1:**
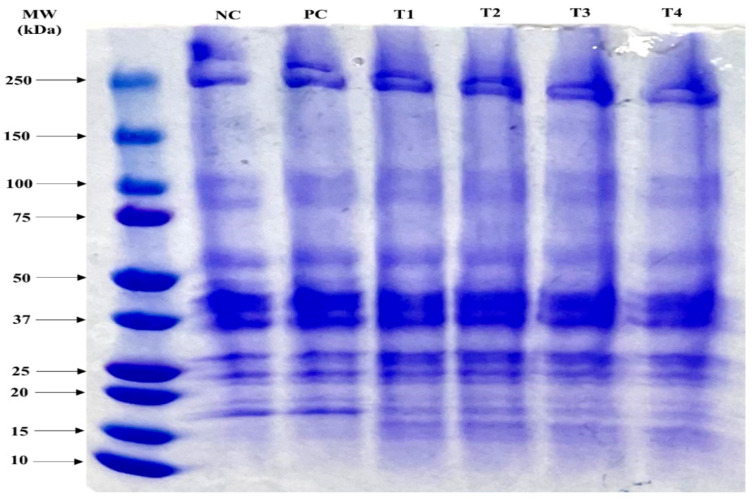
Effects of grafted insect protein and eggshell powder on protein molecular weight distribution of phosphate-free meat emulsion. NC: negative control not containing phosphate, PC; positive control containing 0.15% phosphate, T1: meat emulsion containing 0.10% grafted insect protein (GI), T2: meat emulsion containing 0.1% GI and 0.1% eggshell powder (ES), T3: meat emulsion containing 0.15% GI, T4: meat emulsion containing 0.15% GI and 0.1% ES.

The protein molecular weight distributions of the meat emulsions containing GI and ES are presented in Figure 1. Clear differences between treatments and controls were not observed for high molecular weights (>50 kDa). However, the protein distribution changed in molecular weights less than 25 kDa. This result may have been due to the addition of insect proteins. Entomic proteins can inhibit the formation of stable disulfide bonds between myosin and porcine proteins or can interact with the latter [7,32]. When substituted with an entomic protein for porcine protein, the protein distribution in low molecular weight decreased [32]. Although certain protein bands expected for myosin light chain (<16 kDa) were observed in NC and PC, band intensities < 20 kDa were faint, and protein bands between 20 and 25 kDa of treatments were thicker than those in NC and PC, even though a protein band at 16 kDa was not observed. Therefore, the addition of GI may interact with porcine proteins, especially the myosin light chain, which may affect heat-induced protein gelation and structural characteristics.

### 3.4. Apparent Viscosity

**Figure 2:**
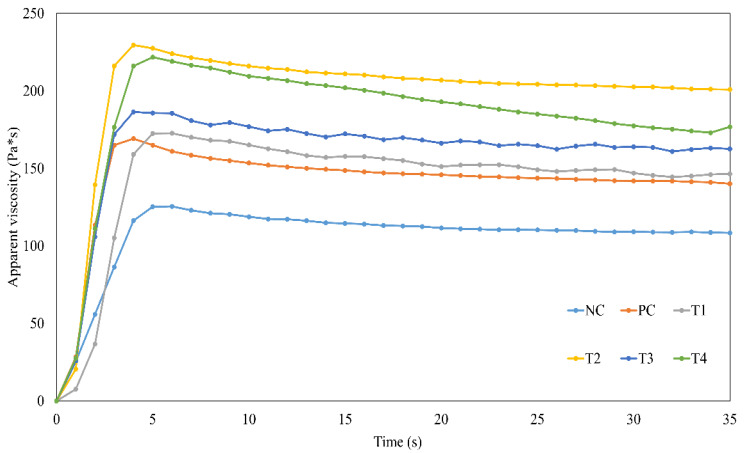
Effects of grafted insect protein and eggshell powder on the texture profile analysis of phosphate-free meat emulsion. NC: negative control not containing phosphate, PC: positive control containing 0.15% phosphate, T1: meat emulsion containing 0.10% GI, T2: meat emulsion containing 0.1% GI and 0.1% ES, T3: meat emulsion containing 0.15% GI, T4: meat emulsion containing 0.15% GI and 0.1% ES.

The apparent viscosities of the meat emulsions containing GI and ES are presented in Figure 2. All treatments and controls showed thixotropic behavior, NC had lower viscosity, and the addition of GI and ES increased the viscosity of the meat emulsion. The surface hydrophobicity of the grafted protein with reducing sugars could be enhanced, and this increased surface hydrophobicity could enhance viscous characteristics [15,31]. In addition, increased protein solubility can affect the viscosity of meat emulsions. T2 and T4 had the highest viscosities, followed by T1 and T3. A highly viscous meat emulsion is correlated with high emulsifying properties [19]. Therefore, T2 had the highest emulsifying properties among all the treatments.

### 3.5. Cooking Loss, Water Holding Capacity, and Emulsion Stability

The cooking loss, water holding capacity, and emulsion stability of the meat emulsions containing GI and ES are presented in Table 4. Although T1 showed no significant difference from NC in cooking loss (*p* > 0.05); other treatments had lower cooking loss values compared with NC (*p* < 0.05), and even T2 and T4 had lower values than PC in cooking loss (*p* < 0.05). Reducing sugars increases the structural flexibility of the grafted protein, and the water binding capacity can be enhanced [15]. Additionally, eggshells can increase the pH value of meat emulsions, and cooking loss might be improved by increasing the pH of the meat emulsion [29]. Although the addition of GI can improve water holding capacity, the addition of 0.15% GI (T3 and T4) showed no significant difference when compared with NC (*p* > 0.05). This result may have been due to the unstable structure of the heated meat emulsion (see Section 3.2 and Section 3.3). When total expressible fluid separation was performed, there was a similar result to cooking loss. NC had the highest value, and PC, T2, and T4 had the lowest values for total expressible fluid separation (*p* < 0.05). When comparing fat separation, T1 and T2 had the lowest values, and other treatments, including controls, showed no significant difference (*p* < 0.05). The addition of GI enhanced water holding capacity and emulsion stability, but excessive addition of GI inhibited the formation of a stable emulsion structure. Therefore, 0.10% GI can be used to enhance the quality characteristics of meat emulsions.

### 3.6. Lipid Oxidation

Initial lipid oxidation should be regulated to extend the shelf life of meat emulsions. In addition, lipid oxidation can be accelerated when the emulsion structure is unstable [33]. The effects of GI and ES on lipid oxidation are presented in Table 4. NC had the highest TBARS value, whereas PC had the lowest TBARS value (*p* < 0.05). This result might be due to the potential chelating effect of phosphate, which could inhibit lipid oxidation of meat emulsions [34,35]. When comparing treatments and controls, the TBARS value of treatments was lower than that of the NC (*p* < 0.05) and higher than that of the PC. The reducing powder of conjugated proteins with reducing sugars can enhance the antioxidant capacity of proteins [36]. In addition, the extracted insect proteins have potential antioxidant capacities [5]. This increase in antioxidant capacity may inhibit lipid oxidation in meat emulsions. When comparing the TBARS values among treatments, the meat emulsion with higher GI had a lower TBARS value, and the addition of ES had a positive effect on lipid oxidation. This result might be due to the antioxidant effect of GI and the meat emulsion structure stabilized by ES.

### 3.7. Texture Profile Analysis

The textural properties of meat emulsions containing GI and ES are presented in Table 5. The highest hardness was observed in PC, and the lowest hardness was observed in T4 (*p* < 0.05), even though the treatments had a lower value than NC (*p* < 0.05). The springiness of the treatments was lower than that of the control, with T4 having the lowest springiness value (*p* < 0.05). The addition of GI and ES might inhibit tight gel formation and cavitated meat emulsions [29,32], with moisture loss collapsing heat-induced meat emulsion [37,38]. When replace meat by mealworm powder, increased replacement induced poor textural properties [9]. Decreased hardness and springiness affected cohesiveness, gumminess, and chewiness. PC had the highest cohesiveness, gumminess, and chewiness values, followed by NC (*p* < 0.05). T3 and T4 had the lowest cohesiveness values (*p* < 0.05). These results suggest that the addition of GI and ES inhibited tight gel formation.

## 4. Conclusions

In this study, insect proteins grafted with palatinose and eggshell powder were applied to a phosphate-free meat emulsion to improve quality properties. Grafted insects increase the pH value, and the typical color of insect powder affects the color value of meat emulsions. Protein solubility, especially myofibillar protein, was improved with the addition of grafted insect protein. Cooking loss did not improve, but water holding capacity, emulsion stability, viscosity, and lipid oxidation improved. Egg shells can improve the quality of meat emulsions containing grafted insect proteins. In terms of textural properties, the hardness and springiness of treatments decreased with the addition of grafted insect protein and eggshells. In this study, except for textural properties, GI and ES could be used as a phosphate replacement. Therefore, further study should focus on the textural properties of a phosphate-free meat emulsion with grafted insect protein.

## Figures and Tables

**Table 1:** Basic formulation of meat emulsion contained grafted insect protein with reducing sugar (GI) and eggshell (ES).

Traits (g/100 g)	NC	PC	T1	T2	T3	T4
Meat	50	50	50	50	50	50
Ice	25	25	25	25	25	25
Fat	25	25	25	25	25	25
NaCl	1.5	1.5	1.5	1.5	1.5	1.5
Phosphate	-	0.15	-	-	-	-
GI	-	-	0.10	0.10	0.15	0.15
ES	-	-	-	0.10	-	0.10

**Table 2:** Effects of grafted insect protein and eggshell powder on pH and color of phosphate-free meat emulsion.

Traits		NC ^(1)^	PC	T1	T2	T3	T4
Raw	pH	5.92 ± 0.01 ^f^	6.12 ± 0.02 ^c^	6.01 ± 0.01 ^d^	6.33 ± 0.03 ^a^	5.97 ± 0.05 ^e^	6.27 ± 0.02 ^b^
	CIE L*	75.89 ± 0.37 ^b^	79.84 ± 0.45 ^a^	74.85 ± 0.92 ^b,c^	73.32 ± 1.51 ^d^	74.11 ± 1.31 ^c,d^	75.27 ± 2.66 ^b,c^
	CIE a*	9.47 ± 0.97 ^a^	8.38 ± 0.33 ^b,c^	7.72 ± 0.56 ^c^	8.58 ± 0.40 ^b^	7.88 ± 0.82 ^b,c^	6.73 ± 0.64 ^d^
	CIE b*	12.38 ± 0.83 ^c^	12.03 ± 0.33 ^c^	13.25 ± 0.33 ^b^	13.97 ± 0.41 ^b^	14.86 ± 1.14 ^a^	13.27 ± 0.74 ^b^
Cooked	pH	6.13 ± 0.01 ^c^	6.20 ± 0.02 ^b^	6.08 ± 0.01 ^d^	6.35 ± 0.04 ^a^	6.09 ± 0.01 ^d^	6.33 ± 0.03 ^a^
	CIE L*	80.45 ± 0.37 ^a^	81.30 ± 0.79 ^a^	77.77 ± 0.67 ^b^	78.35 ± 1.03 ^b^	75.79 ± 1.24 ^c^	76.55 ± 0.98 ^c^
	CIE a*	3.19 ± 0.13 ^a^	2.78 ± 0.16 ^b^	2.54 ± 0.12 ^d^	2.72 ± 0.12 ^b,c^	2.59 ± 0.12 ^c,d^	2.67 ± 0.12 ^c,d,e^
	CIE b*	10.21 ± 0.20 ^d^	9.35 ± 0.21 ^e^	11.45 ± 0.16 ^a,b^	10.88 ± 0.39 ^c^	11.25 ± 0.17 ^b^	11.56 ± 0.30 ^a^

All values are mean ± standard error of three replicates. ^a–f^ Means within a row with different letters are significantly different (*p* < 0.05). ^(1)^ NC: negative control containing no-phosphate, PC: positive control containing 0.15% phosphate, T1: meat emulsion containing 0.10% grafted insect protein (GI), T2: meat emulsion containing 0.1% GI and 0.1% eggshell powder (ES); T3: meat emulsion containing 0.15% GI; T4: meat emulsion containing 0.15% GI and 0.1% ES.

**Table 3:** Effects of grafted insect protein and eggshell powder on protein solubility of phosphate free meat emulsion.

Traits	NC ^(1)^	PC	T1	T2	T3	T4
Total soluble protein (mg/g)	81.88 ± 0.28 ^d^	87.40 ± 1.51 ^c^	90.44 ± 1.21 ^b^	90.2 ± 1.35 ^b^	92.84 ± 2.33 ^a,b^	93.74 ± 2.02 ^a^
Water soluble protein (mg/g)	45.77 ± 1.80	46.94 ± 0.71	47.82 ± 1.70	48.44 ± 1.86	49.00 ± 1.52	49.82 ± 1.51
Salt soluble protein (mg/g)	36.12 ± 1.61 ^d^	40.47 ± 0.83 ^c^	42.62 ± 0.65 ^b^	41.76 ± 0.77 ^b,c^	43.84 ± 1.30 ^a^	43.92 ± 0.76 ^a^

All values are mean ± standard error of three replicates. ^a–d^ Means within a row with different letters are significantly different (*p* < 0.05). ^(1)^ NC: negative control containing no-phosphate, PC: positive control containing 0.15% phosphate, T1: meat emulsion containing 0.10% GI, T2: meat emulsion containing 0.1% GI and 0.1% ES; T3: meat emulsion containing 0.15% GI; T4: meat emulsion containing 0.15% GI and 0.1% ES.

**Table 4:** Effects of grafted insect protein and eggshell powder on cooking loss, water holding capacity (WHC), emulsion stability, and thiobarbituric acid reactive substances (TBARS) of phosphate free meat emulsion.

Traits		NC ^(1)^	PC	T1	T2	T3	T4
Cooking loss (%)		22.82 ± 1.28 ^a^	13.03 ± 1.09 ^c^	20.88 ± 0.42 ^a,b^	10.53 ± 0.84 ^d^	20.10 ± 0.79 ^b^	10.12 ± 1.35 ^d^
WHC (%)		72.28 ± 0.88 ^c^	75.18 ± 1.34 ^a^	76.47 ± 1.51 ^a^	74.56 ± 0.27 ^a,b^	72.65 ± 0.42 ^b,c^	71.27 ± 1.57 ^c^
Emulsion stability	Total expressible fluid separation	23.70 ± 0.80 ^a^	8.00 ± 2.46 ^c^	19.45 ± 1.65 ^b^	7.02 ± 0.38 ^c^	20.08 ± 2.24 ^b^	6.87 ± 1.67 ^c^
	Fat separation	0.98 ± 0.03 ^a^	0.98 ± 0.50 ^a^	0.49 ± 0.01 ^b^	0.50 ± 0.01 ^b^	0.90 ± 0.28 ^a^	0.90 ± 0.01 ^a^
TBARS (mg/kg)		1.49 ± 0.02 ^a^	0.13 ± 0.01 ^f^	0.72 ± 0.01 ^b^	0.41 ± 0.01 ^d^	0.68 ± 0.01 ^c^	0.30 ± 0.01 ^e^

All values are mean ± standard error of three replicates. ^a–f^ Means within a row with different letters are significantly different (*p* < 0.05). ^(1)^ NC: negative control containing no-phosphate, PC: positive control containing 0.15% phosphate, T1: meat emulsion containing 0.10% GI, T2: meat emulsion containing 0.1% GI and 0.1% ES; T3: meat emulsion containing 0.15% GI; T4: meat emulsion containing 0.15% GI and 0.1% ES.

**Table 5:** Effects of grafted insect protein and eggshell powder on texture profile analysis of phosphate free meat emulsion.

Traits	NC ^(1)^	PC	T1	T2	T3	T4
Hardness (kg)	3.39 ± 0.38 ^b^	3.93 ± 0.53 ^a^	1.69 ± 0.22 ^c^	1.19 ± 0.30 ^d^	1.41 ± 0.07 ^c,d^	0.87 ± 0.13 ^e^
Springiness	0.80 ± 0.04 ^a^	0.86 ± 0.04 ^a^	0.65 ± 0.06 ^b^	0.65 ± 0.05 ^b^	0.62 ± 0.08 ^b^	0.54 ± 0.09 ^c^
Cohesiveness	0.67 ± 0.02 ^b^	0.71 ± 0.01 ^a^	0.45 ± 0.05 ^c^	0.38 ± 0.04 ^d^	0.34 ± 0.03 ^e^	0.34 ± 0.03 ^e^
Gumminess (kg)	2.27 ± 0.24 ^b^	2.79 ± 0.36 ^a^	0.77 ± 0.15 ^c^	0.46 ± 0.16 ^d^	0.48 ± 0.05 ^d^	0.30 ± 0.04 ^d^
Chewiness (kg)	1.84 ± 0.27 ^b^	2.39 ± 0.36 ^a^	0.50 ± 0.11 ^c^	0.30 ± 0.10 ^d^	0.30 ± 0.06 ^d^	0.16 ± 0.04 ^d^

All values are mean ± standard error of three replicates. ^a–e^ Means within a row with different letters are significantly different (*p* < 0.05). ^(1)^ NC: negative control containing no-phosphate, PC: positive control containing 0.15% phosphate, T1: meat emulsion containing 0.10% GI, T2: meat emulsion containing 0.1% GI and 0.1% ES; T3: meat emulsion containing 0.15% GI; T4: meat emulsion containing 0.15% GI and 0.1% ES.

## Data Availability

Data are contained within the article.
